# Association of social restrictions with fear of cancer recurrence in hematopoietic stem cell transplantation patients: an exploratory analysis of the potential chain mediation role of illness perception and cognitive emotion regulation

**DOI:** 10.3389/fpsyt.2026.1878087

**Published:** 2026-08-21

**Authors:** Shurong Ran, Chunmei Zhang, Mingyan Liao

**Affiliations:** Department of Hematology, The First Affiliated Hospital of Chongqing Medical University, Chongqing, China

**Keywords:** disease perception, exploratory mediation analysis, fear of cancer recurrence, hematopoietic stem cell transplantation, social restrictions

## Abstract

**Objective:**

To explore the potential association between social restrictions and FCR in patients who underwent HSCT, and preliminarily investigate whether examine whether illness perception and cognitive emotion regulation mediate this association using an exploratory cross-sectional design.

**Methods:**

We retrospectively collected clinical data from 328 patients who underwent HSCT at the Department of Hematology, the First Affiliated Hospital of Chongqing Medical University, between January 2020 and December 2024. After excluding three invalid cases with incomplete core data, 325 patients were included in the analysis. All participants completed the SCS, IPQ-R, CERQ, and FCRI assessments. We constructed a chain mediation model for exploratory analysis. A sensitivity analysis adjusting for age, sex, and transplant type was further conducted to control for confounding.

**Results:**

Of the 325 patients, 172 experienced mild FCR (52.92%), 128 experienced moderate FCR (39.38%), and 25 experienced severe FCR (7.69%). The SCS, IPQ-R, CERQ, and FCRI scores were all positively correlated with each other in patients who underwent HSCT (all *P* < 0.05). Mediation analysis showed that SCS scores were significantly positively associated with IPQ-R scores, with a small effect size (R = 0.135, R²=0.018, *F* = 6.033, *P* = 0.015). The total statistical association value of social restrictions with FCR was 0.816, with a Bootstrap 95% CI (0.640-0.993) excluding 0. The direct association value was 0.780, and the Bootstrap 95% *CI* (0.593~0.966) excluding 0. The hypothesized SCS→IPQ-R→CERQ→FCR chain mediation pathway was not statistically supported. After adjusting for age, sex, and transplant type, all indirect pathways lost statistical significance, while the direct positive association between social restrictions and FCR remained significant (direct effect=0.252, 95%*CI*: 0.028–0.475, *P* = 0.028).

**Conclusion:**

The prevalence of moderate-to-severe FCR in patients after HSCT is 47.08%. This exploratory study suggests that social restrictions are statistically associated with FCR. The pre-specified chain mediation model was not confirmed after adjusting for covariates. The causal direction and clinical significance of the findings remain to be verified by longitudinal studies.

## 1 Introduction

Hematopoietic stem cell transplantation (HSCT) is an important curative method for treating hematological malignancies, aplastic anemia, and other diseases, and can significantly prolong patint survival. However, after transplantation, patients face long-term problems, such as low immune function, risk of complications, and treatment side effects, leading to fear of cancer recurrence (FCR) which is one of the most common psychological troubles among this population (1, 2). Previous studies have shown that the incidence of moderate to severe FCR in patients after HSCT can reach 40% to 60%, posing a serious threat to their physical and mental recovery (3). Therefore, it is of great clinical significance to clarify factors influencing FCR and develop targeted intervention measures. Social constraints refer to the limitations and barriers individuals encounter in social activities, such as social interaction, work, and recreation, due to illness or disability, which are important environmental factors associated with the psychological status of patients after HSCT (4). Previous studies have confirmed a positive correlation between social restrictions and FCR in cancer patients; however, the mechanism of action between the two is not fully understood, and there may be mediating effects of intermediate variables (5). Disease perception is a comprehensive evaluation of an individual’s cognition, emotions, and beliefs about their disease (6). It has been proposed that social constraints and negative illness perception may be associated with the adoption of negative cognitive emotion regulation strategies, which may, in turn, be linked to heightened concerns about disease recurrence and increased FCR. Existing research has mostly explored the pairwise relationship between social constraints, disease perception, cognitive emotional regulation, and FCR separately, and the chain reaction pathway between the three has not yet been clarified (7, 8). Accordingly, this study proposed an exploratory hypothesis that illness perception and cognitive emotion regulation may play a chain mediating role in the association between social constraints and FCR among patients after HSCT. We conducted a cross-sectional study with 325 patients to preliminarily test this hypothesis and explore the interrelationships among variables, aiming to provide reference evidence for clinical FCR intervention.

## 2 Data and methods

### 2.1 Research object

We retrospectively recruited 328 consecutive patients who underwent HSCT at the Department of Hematology, the First Affiliated Hospital of Chongqing Medical University, from January 2020 to December 2024.

The inclusion criteria were as follows: (1) patients with hematological diseases clinically diagnosed as requiring HSCT, including leukemia, lymphoma, aplastic anemia, and other related disorders; (2) patients who had completed HSCT and survived for ≥1 month after transplantation; (3) availability of complete clinical data; and (4) completion of scale assessments during follow-up 1–3 months after HSCT. The exclusion criteria were as follows: (1) concurrent diagnosis of other malignant tumors; (2) uncontrolled severe post-transplant complications; (3) severe organ dysfunction, including hepatic or renal dysfunction; (4) presence of central nervous system diseases or psychiatric disorders; and (5) cognitive impairment or language communication difficulties that could interfere with the completion of the survey.

After screening, 3 cases with incomplete key scale data were excluded, resulting in a final sample of 325 patients. No imputation was performed for missing data, and only cases with complete data for all core variables were included in the analyses. The cohort included both autologous and allogeneic HSCT recipients.

This study was approved by the Ethics Committee of the First Affiliated Hospital of Chongqing Medical University (approval No. 2026-0236-01). During the study, participants and their families provided informed consent through telephone follow-up, WeChat communication, or written signatures. All procedures were performed in accordance with the Declaration of Helsinki.

### 2.2 Research tool

#### 2.2.1 Social restriction scale

The SCS scale developed by Lepore et al. (9) was used, which includes 15 items rated on a scale of 1-4 (1=completely unrestricted, 4=severely restricted), with a total score of 15–60 points. The higher the score, the higher the patient’s level of social restriction is. The scale has been validated in Chinese among cancer patients in China and has good reliability and validity. In this study, the Cronbach’s alpha coefficient of the scale was 0.823.

#### 2.2.2 Disease perception questionnaire (IPQ-R)

The Moss-Morris et al. (10) revised Disease Perception Questionnaire (IPQ-R) was adopted. This scale consists of 38 items and is divided into seven dimensions: disease identity, disease course cognition, treatment control, personal control, disease consequences, cause attribution, and emotional expression. In this study, the negative dimensions most closely related to FCR - the disease consequences dimension (6 items) and the emotional expression dimension (6 items) - were selected, totaling 12 items. A 5-point rating scale (1 = completely disagree, 5 = completely agree) was used, with a score range of 12 to 60. The higher the score, the stronger the negative disease perception of the patients. The Chinese version of IPQ-R has been validated in breast cancer patients,and has good reliability and validity (11). In this study, the Cronbach’s α coefficient of this part was 0.857.

#### 2.2.3 Cognitive emotion regulation questionnaire

The CERQ was developed by Garnefski et al. (11). The full version contains 36 items across 9 dimensions. Rationale for dimension selection: this study aimed to explore the negative psychological pathway associated with FCR. Rumination, catastrophizing, and self-blame are three well-documented maladaptive cognitive emotion regulation strategies consistently linked to increased psychological distress in cancer patients. Thus, these three negative dimensions (12 items in total) were selected to measure negative cognitive emotion regulation. Items are rated on a 5-point scale (1=almost never, 5=almost always), with scores ranging from 12 to 60. A higher dimension score indicates a greater tendency to adopt negative cognitive emotion regulation strategies. The Chinese version of this scale is widely used in Chinese cancer patients with good reliability and validity. In this study, the Cronbach’s α coefficient for the selected dimensions was 0.839.

#### 2.2.4 Cancer recurrence fear scale (FCRI)

The FCRI scale developed by Lebel et al. (12) was used. It includes 42 items and is divided into five dimensions: fear, triggering factors, functional impairment, seeking assurance, and avoidance. The scale is rated on a scale of 0-4 (0=never, 4=always), with a total score of 0–168 points. According to the FCRI grading standard established by Lebel et al. (13), FCR is classified into mild (0–29 points), moderate (30–59 points), and severe (≥60 points). This grading standard has been verified by the ROC curve and shows good sensitivity and specificity. The incidence of moderate to severe FCR was calculated as (number of moderate cases+number of severe cases) divided by the total number of cases multiplied by 100%. The scale has been localized and validated for reliability and validity in patients after HSCT in China. In this study, the Cronbach’s alpha coefficient of the scale was 0.886.

#### 2.3 Data collection methods

Based on the hospital’s electronic medical record system and hematology follow-up database, we retrieved a list of patients who received HSCT between January 2020 and December 2024 and preliminarily screened potential research subjects who met the inclusion criteria. Two researchers independently reviewed the complete medical records of the candidate patients (inpatient medical records, outpatient follow-up records, and laboratory test reports), strictly followed the inclusion and exclusion criteria for the final screening, and reached a consensus through group discussions on cases with inconsistent screening results. We extracted the clinical data of eligible patients and completed standardized data extraction forms, and simultaneously collected raw data of scale questionnaires completed by patients during post-transplant routine follow-up. Cases with missing core scale data that could not be supplemented by follow-up records were excluded from the analysis. Finally, three invalid cases were excluded, and 325 patients were included in the final analysis.

### 2.4 Statistical methods

SPSS 26.0 statistical software and the PROCESS plugin (version 3.5) were used for data analysis. Measurement data are described using $\left(\bar{x}\pm s\right)$ deviation, while count data are described using example (%). The Shapiro-Wilk test was used to assess the normality of the quantitative variables. The variance inflation factor (VIF) was calculated to test for multicollinearity among the independent variables, with a VIF < 5 indicating no severe multicollinearity. Pearson’s correlation analysis was conducted to explore the bivariate associations among social constraints, illness perception, cognitive emotion regulation, and FCR.

Model 6 of the PROCESS macro was used to conduct exploratory chain mediation analysis. First, an unadjusted baseline model was constructed for preliminary exploration. Then, a sensitivity analysis was performed by including age, sex, and transplant type as covariates in all three regression equations of the mediation model to control for demographic and clinical confounding. The bias-corrected Bootstrap method (5000 resampling iterations) was used to estimate 95% confidence intervals (CIs) for mediation effects. An indirect effect was considered statistically significant if its 95% CI did not contain 0. P values are not reported for indirect effects, as Bootstrap CIs are the standard and appropriate criterion for inference. The significance level was set at α=0.05 (two-tailed).

## 3 Results

### 3.1 General information of 325 patients

Among the 325 patients, 183 (56.31%) were male and 142 (43.69%) were female. The patients’ ages ranged from 18 to 65 years, with a mean age of 42.36 ± 11.25 years. Regarding transplantation type, 158 patients (48.62%) underwent autologous HSCT and 167 (51.38%) underwent allogeneic HSCT. The underlying diseases included leukemia in 176 patients (54.15%), lymphoma in 98 patients (30.15%), and other hematological diseases in 51 patients (15.70%).

### 3.2 Scores on various patient scales

Among patients who underwent HSCT, the mean scores were 37.25 ± 5.30 for the SCS, 45.62 ± 4.64 for the IPQ-R, 36.21 ± 5.62 for the CERQ, and 62.45 ± 9.59 for the FCRI. Regarding FCR severity, 172 patients (52.92%) had mild FCR, 128 (39.38%) had moderate FCR, and 25 (7.69%) had severe FCR. The proportion of patients with moderate-to-severe FCR was 47.08%.

### 3.3 Correlation analysis between variables

The SCS, IPQ-R, CERQ, and FCRI scores were positively correlated (*P* < 0.05), as shown in **Table 1**. All variables had VIF values<5, indicating no severe multicollinearity, and all variables were eligible for inclusion in the regression model.

**Table 1:** Correlation analysis of variables.

Variable	SCS	IPQ-R	CERQ	FCRI
SCS	1	–	–	–
IPQ-R	0.135^*^	1	–	–
CERQ	0.395^**^	0.139^*^	1	–
FCRI	0.451^**^	0.306^**^	0.170^*^	1

* *P* < 0.05; ^**^P < 0.001.

### 3.4 Mediation effect analysis

#### 3.4.1 Unadjusted baseline model

**Figure 1:**
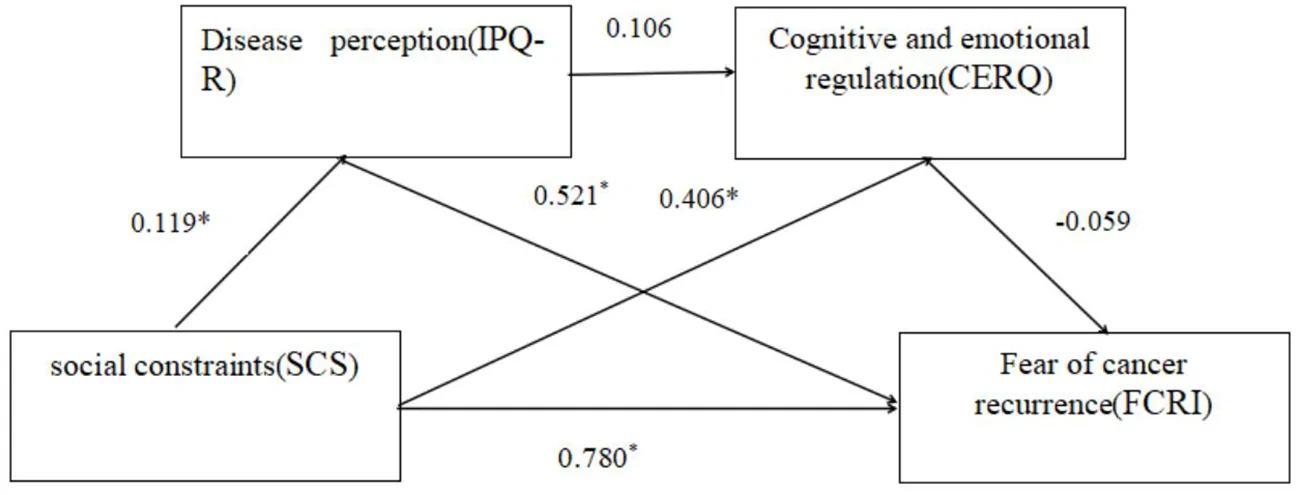
Chain mediation effect model of social restrictions on cancer recurrence fear. ^*^P < 0.05.

With SCS score as the independent variable (X), FCR (FCRI score) as the dependent variable (Y), illness perception (IPQ-R score) as mediator 1 (M1), and cognitive emotion regulation (CERQ score) as mediator 2 (M2), we conducted an exploratory chain mediation analysis. The core results showed that: SCS score was significantly positively associated with IPQ-R score (β=0.119, *t* = 2.456, *P* = 0.015, 95%*CI*: 0.024–0.214), with a small effect size (R = 0.135, R²=0.018, *F* = 6.033, *P* = 0.015).SCS score was significantly positively associated with CERQ score, while the association between IPQ-R score and CERQ score was not statistically significant. The overall model for CERQ was statistically significant (R = 0.404, R²=0.163, *F* = 31.403, *P* < 0.001). Both SCS and IPQ-R were significantly positively associated with FCR. The overall model was statistically significant (R = 0.515, R²=0.265, *F* = 38.642, *P* < 0.001). The total association of social constraints with FCR was 0.816 (Bootstrap 95%*CI*: 0.640–0.993), and the direct association was 0.780 (Bootstrap 95%*CI*: 0.593–0.966). The indirect effect of the SCS→IPQ-R→FCR pathway was 0.062 (Bootstrap 95%*CI*: 0.003–0.153). The indirect effects of the SCS→CERQ→FCR pathway and the SCS→IPQ-R→CERQ→FCR chain pathway both contained 0 in their 95% CIs, indicating no statistical significance. The hypothesized chain mediation pathway was not supported. Detailed results are shown in **Table 2** and **Figure 1**. Notably, some statistically significant pathways had relatively small effect sizes, suggesting that the statistical associations observed should be interpreted with caution regarding their clinical relevance.

**Table 2:** Total, direct and indirect effects in unadjusted and adjusted models.

Effect pathway	Unadjusted model		Adjusted model (age, sex, transplant type)	
	Effect value	Bootstrap 95%*CI*	Effect value	Bootstrap 95%*CI*
Total effect	0.816	0.640–0.993	0.189	−0.036–0.413
Direct effect	0.780	0.593–0.966	0.252	0.028–0.475
Total indirect effect	0.036	−0.074–0.148	−0.063	−0.162–0.025
Ind1: SCS→IPQ-R→FCRI	0.062	0.003–0.153	−0.047	−0.127–0.021
Ind2: SCS→CERQ→FCRI	−0.024	−0.122–0.061	−0.009	−0.067–0.042
Ind3: SCS→IPQ-R→CERQ→FCRI	−0.001	−0.005–0.004	−0.007	−0.027–0.002

Significance of indirect effects is determined by whether the 95% Bootstrap *CI* excludes 0.

#### 3.4.2 Adjusted sensitivity analysis (controlling for age, sex, transplant type)

After adjusting for age, sex, and transplant type, the results were as follows:

1. Path to M1 (IPQ-R): SCS was significantly *negatively* associated with IPQ-R (coeff=−0.2216, *P* = 0.0003, 95%CI: −0.3411 to −0.1021). Transplant type had the strongest independent association with illness perception (standardized *β* = 0.3641, *P* < 0.001). The overall model was significant (R = 0.4462, R²=0.1991, *F* = 19.8913, *P* < 0.0001).
2. Path to M2 (CERQ): Neither SCS (coeff=0.0298, *P* = 0.6732) nor IPQ-R (coeff=−0.0923, *P* = 0.1487) was significantly associated with CERQ. Transplant type and sex remained significant predictors. The overall model was significant (R = 0.5399, R²=0.2915, *F* = 26.2504, *P* < 0.0001).
3. Path to Y (FCRI): SCS remained significantly positively associated with FCRI (direct effect=0.2515, *P* = 0.0276, 95%*CI*: 0.0280–0.4750). IPQ-R was positively associated with FCRI (coeff=0.2118, *P* = 0.0401), while CERQ was negatively associated with FCRI (coeff=−0.3166, *P* = 0.0005). Sex and transplant type also showed significant independent associations. The overall model was significant (R = 0.6116, R²=0.3741, *F* = 31.6766, *P* < 0.0001).

For indirect effects: Total indirect effect: −0.0629 (Bootstrap 95%*CI*: −0.1623 to 0.0251), not significant. Ind1 (SCS→IPQ-R→FCRI): −0.0469 (Bootstrap 95%CI: −0.1273 to 0.0214), not significant. Ind2 (SCS→CERQ→FCRI): −0.0094 (Bootstrap 95%*CI*: −0.0668 to 0.0422), not significant. Ind3 (chain pathway SCS→IPQ-R→CERQ→FCRI): −0.0065 (Bootstrap 95%*CI*: −0.0266 to 0.0023), not significant.

In summary, after controlling for demographic and clinical covariates, all indirect mediation pathways lost statistical significance, while the direct positive association between social restrictions and FCR remained robust.

## 4 Discussion

### 4.1 Overall core findings

This study focused on 325 HSCT patients and systematically explored the relationships between social constraints, disease perception, negative cognitive emotion regulation, and FCR. The core finding was that the pre-specified chain mediation hypothesis (social constraints → illness perception → cognitive emotion regulation → FCR) was not supported by the data. After further adjusting for age, sex, and transplant type in sensitivity analysis, all indirect mediation pathways lost statistical significance, and only the direct positive association between social restrictions and FCR remained stable. This result indicates that the association between social restrictions and FCR in HSCT patients is largely confounded by clinical and demographic characteristics, and the hypothesized psychological mediation pathway is not supported after controlling for these covariates. This study found that the incidence of moderate-to-severe FCR was higher (47.08%), consistent with the reported incidence of 40% to 60% in previous studies (14, 15). Further confirmation shows that FCR is a high incidence of psychological distress in patients after HSCT after, and clinical screening and intervention of FCR must be included in the normalized management, rather than just focusing on patients’ physical recovery.

### 4.2 Bivariate associations among variables

The correlation analysis showed a positive correlation between SCS, IPQ-R, CERQ and FCRI scores. This result indicates that the direction of the correlation between variables is completely consistent with the theoretical hypothesis: the more social activity restrictions patients feel, the stronger their negative cognitive evaluation of the disease, and the more likely they are to adopt negative coping strategies such as contemplation, catastrophizing, and self-blame, leading to a stronger fear of recurrence. FCR is not a simple emotional response but a comprehensive psychological stress response closely related to the social environment, disease cognition, and emotional coping styles. This suggests that clinical attention should not only focus on physical rehabilitation but also include FCR screening and intervention in routine management of HSCT. This result is consistent with the stress cognitive assessment theory: external stressors (social constraints) are associated with psychological stress outcomes through individual cognitive appraisal processes (disease perception) and emotional coping strategies (cognitive emotional regulation) (16, 17). The significant correlation between pairwise variables provides a basis for subsequent mediation effect tests and indicates that social constraints transmit risk through a series of psychological mediation variables.

### 4.3 Direct and indirect associations of social constraints with FCR

In the unadjusted model, social constraints were significantly directly associated with FCR, which is consistent with existing research. After HSCT, patients commonly experience reduced social interaction, weakened family and social support, and impaired social roles due to immune deficiency, activity restrictions, changes in appearance, and other post-transplant conditions. As a persistent environmental stressor, social constraints may directly reduce patients’ sense of security and control, maintain a long-term state of vigilance and concern, and thus be associated with higher FCR levels (18).This result suggests that reducing social restrictions, expanding social participation, and rebuilding social roles are among the most direct and effective ways to alleviate FCR in patients after HSCT. However, after adjusting for covariates, the mediating effect of illness perception was no longer statistically significant. This suggests that the association between social constraints and illness perception observed in the bivariate analysis may be largely driven by clinical characteristics such as transplant type. Allogeneic HSCT recipients usually face longer isolation periods, higher complication risks, and greater uncertainty about prognosis, which simultaneously increase perceived social restrictions and negative illness perception. After controlling for transplant type, the independent association between social constraints and illness perception even reversed direction, which may reflect a psychological adaptation phenomenon: patients with more severe social restrictions may gradually form more realistic cognitive expectations of the disease under long-term adaptation, rather than increasingly negative perceptions. This unexpected finding needs to be verified in larger cohorts with stratified analysis by transplant type (19–21).

### 4.4 Explanations for the unsupported chain mediation pathway

The independent mediating effect of negative cognitive emotion regulation was not established in this study, and the chain mediation pathway was not statistically supported. Although correlation analysis showed a positive correlation between CERQ and FCR, the association between CERQ and FCR became non-significant and even reversed direction after controlling for social constraints and illness perception. Several possible explanations are proposed for this negative finding. First, the specific psychological adaptation characteristics of the HSCT population: unlike patients with solid tumors, patients after HSCT undergo intensive treatments such as myeloablative chemotherapy and prolonged isolation in laminar flow wards. It has been hypothesized that negative cognitive emotion regulation in this population may serve as an adaptive coping strategy for “preparing for the worst outcome”, rather than a pure risk factor for increased fear. Patients who mentally accept the possibility of recurrence in advance through catastrophic thinking may experience less realistic fear of recurrence in the short term. However, this explanation remains speculative and is not supported by data from the current study. Further longitudinal and qualitative studies are needed to verify this hypothesis.Second, there is a potential suppression effect between variables. The standardized regression coefficient showed that the association between social constraints and the CERQ was much stronger than that between illness perception and the CERQ, suggesting that the effect of illness perception on cognitive emotion regulation may be suppressed by the strong effect of social constraints, leading to a non-significant IPQ-R→CERQ pathway. Third, there are limitations in the dimension selection. This study only included negative dimensions of cognitive emotion regulation and did not examine the positive dimensions, which may not fully capture the role of cognitive emotion regulation in the pathway. This may also contribute to the negative results of the chain pathway.

Overall, these findings suggest that the role of cognitive emotion regulation in FCR among patients after HSCT is not a simple linear mediation relationship. Future studies should further explore its potential moderating effects or incorporate positive cognitive emotion regulation into the model (22–24).

### 4.5 Limitations

The study has a cross-sectional design, which can only infer statistical associations between variables and cannot determine strict causal relationships. In the future, prospective cohort studies should be conducted to dynamically measure variables at multiple time points after HSCT to verify the stability and causal direction of the model. The cohort included both autologous and allogeneic HSCT recipients, who differed substantially in terms of treatment intensity, complication risk, and prognosis. This clinical heterogeneity may affect the generalizability and interpretability of our results. Future studies should conduct stratified analyses based on transplant type. Although we adjusted for age, sex, and transplant type in the sensitivity analysis, several other clinically important variables that may influence FCR were not adjusted for, including graft-versus-host disease, relapse status, active infection, immunosuppressive therapy, corticosteroid exposure, conditioning intensity, donor type, psychiatric history, anxiety/depression status, and social support. Residual confounding cannot be excluded, which limits the interpretability of the regression and mediation results. Only the negative dimensions of the IPQ-R and CERQ were selected, and the full scales were not used, resulting in an incomplete model. Some statistically significant pathways had relatively small effect sizes and low explanatory power, and their clinical significance remains to be confirmed. Future research could adopt a longitudinal tracking design, measure various variables at multiple time points after HSCT, incorporate the complete IPQ-R and CERQ scales, and explore other possible mediating variables to gain a more comprehensive understanding of the psychological pathways associated with FCR in patients after HSCT.

In summary, the prevalence of moderate-to-severe FCR in patients after HSCT is 47.08%. This exploratory study suggests that social restrictions are statistically associated with FCR. The pre-specified chain mediation model was not confirmed after adjusting for covariates. The causal direction and clinical significance of the findings remain to be verified by longitudinal studies.

## Data Availability

The original contributions presented in the study are included in the article/supplementary material. Further inquiries can be directed to the corresponding author.
